# Improving Fingerprint-Based Positioning by Using IEEE 802.11mc FTM/RTT Observables

**DOI:** 10.3390/s23010267

**Published:** 2022-12-27

**Authors:** Israel Martin-Escalona, Enrica Zola

**Affiliations:** Network Engineering Department, Universitat Politecnica de Catalunya, 08034 Barcelona, Spain

**Keywords:** positioning, location, IEEE 802.11mc, RTT, Wi-Fi, positioning error, fingerprinting, RSS, machine learning, under-coverage, scalability

## Abstract

Received signal strength (RSS) has been one of the most used observables for location purposes due to its availability at almost every wireless device. However, the volatile nature of RSS tends to yield to non-reliable location solutions. IEEE 802.11mc enabled the use of the round trip time (RTT) for positioning, which is expected to be a more consistent observable for location purposes. This approach has been gaining support from several companies such as Google, which introduced that feature in the Android O.S. As a result, RTT estimation is now available in several recent off-the-shelf devices, opening a wide range of new approaches for computing location. However, RTT has been traditionally addressed to multilateration solutions. Few works exist that assess the feasibility of the RTT as an accurate feature in positioning methods based on classification algorithms. An attempt is made in this paper to fill this gap by investigating the performance of several classification models in terms of accuracy and positioning errors. The performance is assessed using different AP layouts, distinct AP vendors, and different frequency bands. The accuracy and precision of the RTT-based position estimation is always better than the one obtained with RSS in all the studied scenarios, and especially when few APs are available. In addition, all the considered ML algorithms perform pretty well. As a result, it is not necessary to use more complex solutions (e.g., SVM) when simpler ones (e.g., nearest neighbor classifiers) achieve similar results both in terms of accuracy and location error.

## 1. Introduction

In the era of information, location is perceived as a highly valuable piece of information that can be exploited by location services to drive users to their favorite places. global navigation satellite systems (GNSSs) have been used for providing independent, accurate and ubiquitous location services. However, their use is compromised in scenarios where the user has nonline of sight (NLOS) to the satellites (e.g., indoor environments and urban canyons). Currently, no location system has been able to provide the same coverage and accuracy in indoor scenarios as GNSS can outdoors, especially when dual-band devices are used. Several technologies have been proposed to address this problem, including the ultrawide band (UWB) [1], radio frequency identification (RFID) [2], Bluetooth low energy (BLE) beacons [3] or magnetic field sensing [4]. These technologies involve the deployment of custom hardware for location only purposes, which limits their availability to specific services that require very accurate positions at any cost.

Large efforts have been made to leverage the large availability of data from regular communication networks that can be converted into a position. An appealing technology that can be exploited for location purposes is IEEE 802.11, since it offers a good alternative in terms of accuracy, precision and cost compared to similar systems [5,6]. Among different approaches that are used to convert an IEEE 802.11 measurement into a position [7], fingerprinting seems to be the best candidate as it is a passive solution and does not require line of sight (LOS) to the access points (APs). In the past few years, a lot of interest raised among researchers on the use of the received signal strength (RSS) in fingerprint-based location estimation algorithms, since RSS measurements can be gathered easily by any commercial/consumer off-the-shelf (COTS) smartphone without any additional hardware equipment. However, RSS readings are known to be highly variable due to changes in the environment or to multipath fading [8].

Recently, the use of IEEE 802.11mc round trip time (RTT), which is also known as a fine time measurement (FTM), as an observable in fingerprinting solutions has been suggested [9,10] and is expected to provide more stable measurements compared to using the RSS [11]. However, so far, the RTT has been typically used as an observable for multilateration purposes [7].

In the following, the motivation behind this work is illustrated and the main contributions of this paper are highlighted.

### 1.1. Motivation

Fingerprinting consists of two stages: (1) the construction of an offline fingerprint database with a given observable from the APs in sight; (2) the matching of the data observed from the user’s device with the data stored in the fingerprint database to infer the most likely position of the device. Despite providing good to excellent performance, fingerprinting comes with two main issues that should be addressed: (1) the database construction and maintenance, and (2) the variability in the observations. The database construction often becomes a costly task in terms of time and resources, as it requires defining a dense grid of points covering the area where the location system is going to be deployed, and then conducting a survey to collect a large amount of measurements at each point, so that the fingerprints can be computed. Moreover, further changes in the environment require the database to be updated and consequently fingerprints to be eventually retaken.

Several approaches have been proposed to address these issues. They can be classified in two groups: database estimation and collaborative data collection. The former is addressed at skipping the construction of the fingerprint database by estimating the values that should be observed in a discrete set of virtual reference points (VRPs) and then interpolate the remaining ones until obtaining the desired precision. This approach is taken by the authors in [12], who proposed estimating the fingerprints at the VRPs through a nonzero mean Gaussian process regression trained from a few actual measurements made at the APs. Similarly, a basic radio map is built in [13] to subsequently expand it by using the Biharmonic Spline Interpolation (BSI) method. The second group consists of approaches that dynamically build the fingerprint database from measurements reported by the users of the location system. Since these measurements come from several sources and persists on time, the database remains updated anytime as long as there are enough users. For instance, the authors in [14] propose a system to build crowd-sensed radio maps, where a particle filter coupling inertial sensors and a multivariate Gaussian fingerprinting is placed on top to enhance the accuracy of crowd-sourcing indoor positioning. Likewise, the authors in [15] propose a method to transfer knowledge from the old RSS-based radio maps to a new one by minimizing the Wasserstein distance. In this way, the data distribution in the new map can be better matched with the old one, thus improving the positioning performance.

The other issue that fingerprint systems need to address is coping with the variability in the observations. Historically, the RSS has been the preferred observable used to create the fingerprint database [16,17] since it can be passively measured by any COTS Wireless Fidelity (Wi-Fi) device. However, RSS-based fingerprinting is vulnerable to environmental dynamics [18], thus compromising its scalability and deployment. The authors in [19] observed that, the more the APs in the testbed space, the higher the efficiency of fingerprint-based algorithms. However, because collecting RSS measurements is time and effort consuming, the localization cost increases with the number of APs [20]. This is especially problematic as the environment changes over time. As a result, it is necessary to periodically update the database, which requires extra time, effort, and cost.

Most recent solutions suggest to use the channel state information (CSI) as input to fingerprinting solutions [21,22,23], as it provides richer and more reliable data that pave the way for better data matching at the location stage. However, obtaining the CSI needs to be supported by hardware, which generally does not apply to COTS devices [24] and hence limits a global adoption of CSI.

Ranging can also be estimated by measuring the time-of-flight (TOF) of a signal, i.e., the time a frame takes from a device to an AP, or the RTT, i.e., the time it takes from the device to an AP and back. However, very precise time measurements are required in order to obtain accurate ranging estimations. As a communication network, the IEEE 802.11 technology did not provide a way to compute such measurements from the very beginning, since they were not necessary for the network operation. This feature has been made available since IEEE released the IEEE 802.11mc standard in 2016 [25], as it allows accurate RTT measurements.

For a few years, only some devices from specific manufacturers included such enhancements, but positioning capabilities were mostly left aside, though. Finally, in 2018, Google announced the support of positioning through FTM in Android devices from version 9 (Pie), and network manufacturers (e.g., Intel, Aruba) started supporting the technology in their devices. The list of available devices supporting the IEEE 802.11mc feature is growing and growing [26]. This new scenario, with native support for accurate time measurements in Wi-Fi networks, have boosted the development of location solutions based on multilateration with FTM measurements. For this approach to work, the positions where the APs are settled must be known. This information is normally not provided by the APs in private networks. The authors in [27] propose a double-iteration approach to mitigate this issue, where the APs positions are computed in a first stage by using a GNSS solution. Then, in a second stage, these positions feed a hybrid GNSS/FTM-based Wi-Fi position system to compute the user’s position, at the cost of inheriting the errors that come with APs positions. Moreover, RTT measurements are very sensitive to NLOS conditions [28], which can lead to aberrant errors in the computed position [29,30].

Using traditional RSS observables in fingerprinting systems has some clear advantages over RTT. Firstly, RSS data are available in almost every COTS wireless device, whereas RTT availability requires network devices to support the IEEE 802.11mc technology. Secondly, RTT estimations require some location traffic (i.e., FTM frames) to be injected in the network, which reduces the throughput available for regular data services. The larger the amount of RTT estimates, the lower the bandwidth left for general data purposes. On the other hand, RTT observables are expected to be less sensitive to the scenario than RSS-based ones. Thus, the RTT tends to be more stable than the RSS. Moreover, slight environment changes that could severely impact the RSS are often less noticeable in terms of the RTT.

Accordingly, RTT and RSS observations present several features that look complementary, as shown in Table 1. Thus, coupling several observables could overcome those issues, as recently suggested. The authors in [9] propose an RTT-RSS fingerprinting model where positions are estimated based on the similarity between real-time sensed RTT and RSS measurements against the fingerprint map data previously collected during the offline phase. According to the authors in [9], this similarity approach allows the location system to overcome the typical indoor environment localization challenges, such as multipath interference and NLOS-related transmission problems. In [10], the authors propose an indoor fingerprinting system based on deep neural networks. This system leverages both the RTT and RSS with a model that addresses the multipath, NLOS, signal attenuation, and interference challenges of the indoor environments. Despite coupling the RTT and the RSS measurements provides good results, the authors in [10] state that the benefits from RSS observations are scarce. However, there are no data on how the frequency band or the use of simpler (and cheaper) machine learning algorithms could impact an RTT-based fingerprinting system.

Fixing the user’s position in a fingerprinting system is understood as a classification problem. Once the measurements of the neighboring APs have been collected, they are compared with the fingerprints in the database to find the best matching entry (i.e., the most likely position where the station (STA) is expected to be). Several classification algorithms that are available in the literature can be used for positioning [31]. In the case of RSS fingerprinting solutions, it is well known that the nearest neighbor (NN) provides the best trade-off between positioning error, complexity, and power consumption [32]. However, it is not clear whether the same applies to other observables, such as the RTT [7]. In this context, this paper investigates employing the RTT, instead of the RSS, as an observable to construct the fingerprint database for positioning purposes. The higher stability of the RTT is expected to provide more accurate and precise positions compared to the use of the RSS.

The aim of this paper is to fill the gap that still exists in the literature. In [33], which was published in June 2022, the authors surveyed 119 papers on machine learning (ML) algorithms applied to indoor positioning. In 114 of the 119 papers published between 2016 and 2021, the metric used for positioning was the RSS. In fact, the survey [33] does not mention any work that uses the FTM by assessing the performance of several state of the art (SoA) ML classifiers when applied to an RTT-based fingerprinting solution. The results shown in this paper are also compared with SoA RSS-based fingerprinting approaches. Moreover, since the FTM-procedure needs the Wi-Fi STA and the AP to explicitly exchange special messages in order to obtain the timestamps for the distance estimation, the more the APs to be pinged, the higher the overhead and, thus, the lower the available bandwidth for other users’ data exchange. In order to address this scalability issue that affects any positioning approach based on FTM observables, the impact on the performance of the positioning algorithm when using a smaller number of APs is also assessed, both in the case of the RTT-based and of the SoA RSS-based fingerprint approaches. Notice that the same solutions introduced above might also be applied here to alleviate the database construction. However, this paper is aimed at assessing the impact of using the RTT in fingerprinting positioning systems. Therefore, efforts on overcoming other fingerprinting issues, such as reducing the cost of database construction, are postponed to further stages, as long as RTT data reveal being valuable enough to be used in fingerprinting positioning systems.

### 1.2. Contributions

In this paper, an RTT-based fingerprint radio map is presented, which was obtained through a measurement campaign in May 2021. The radio map stores the ranges from IEEE 802.11mc compatible APs located in the auditorium of our university. To the best of the authors’ knowledge, this paper is the first in the literature at studying whether the IEEE 802.11mc FTM can contribute as an observable to fingerprint-based positioning [7,33], rather than providing a complete location system definition. The raw performance of six of the most popular supervised learning techniques [34] when applied to FTM observables is assessed and compared to what RSS-based fingerprinting solutions would achieve under the same conditions. The quality of the match is quantified according to a distance model, which works as a loss function: the larger the value, the worse the position estimation. The use of the FTM as an observable is thus validated, either to be coupled to already proposed RSS-based systems or as the main observable for further fingerprint solutions.

The main contributions of this paper are as follows:

1. The accuracy and stability of the FTM observables in the fingerprint database are discussed;
2. The accuracy of different classification algorithms is assessed when positioning a STA using the FTM procedure defined in the IEEE 802.11mc standard. The accuracy is always higher than 99%, whatever the AP vendor working in any of the 5 GHz channels assessed in this study;
3. The performance of the FTM-based positioning approach is compared with the RSS-based one, both in terms of accuracy and precision of the position estimations, showing that the former outperforms the latter for all the AP brands working in the 5 GHz channels. In the worst case, the mean absolute error of the RSS-based positioning can be up to 10x higher than the RTT-based one;
4. The impact of using measurements from a smaller number of APs is studied in the 5 GHz band, both for RTT and RSS observations. While decreasing the number of APs is known to have a negative impact on the RSS- fingerprint-based positioning [19], to the best of the authors’ knowledge, there is no study analyzing its impact on RTT-based fingerprinting. However, since RTT estimations require specific frames being exchanged in the shared medium, the available throughput may be constrained when multiple users are trying to locate themselves; thus, assessing the performance when a lower number of APs are involved in the measurements is of key importance.

This paper is organized as follows: First, the methodology used to gather the RTT measurements and the scenario where the radio map was constructed are described in Section 2. Section 3 presents the resulting dataset and compares some statistics, especially focusing on the higher deviations in the RSS measurements compared to those in the RTT; in addition, a brief discussion of the ranging error observed in the collected data are provided, which indicates poor accuracy for applying multilateration methods with RTT observations. Instead, a fingerprinting approach is taken in this paper, which enables the conversion of the RTT measurements reported by the STA into positions, by matching them with the RTT fingerprints in the database (i.e., traditional ML models in [24]). The performance of this positioning model is assessed in Section 4, where several SoA classification algorithms are compared, while considering different layouts and frequency bands. In order to assess the accuracy of the classifiers with both RTT- and RSS-based fingerprinting, testing is performed at the locations where the measurements were gathered, which is in line with other recent works [34]. Validation at other locations is out of the scope of this paper, as it would require also defining and evaluating a post filtering stage, which is necessary in order to be able to position the user in any point inside the grid (e.g., centroid-based approach [35], particle filter [36], regression ML models [37], etc.). The final remarks in Section 5 conclude the paper, while Section 6 highlights some open issues that are worth further investigation in the near future.

## 2. Measurement Campaign

### 2.1. Methodology

**Figure 1:**
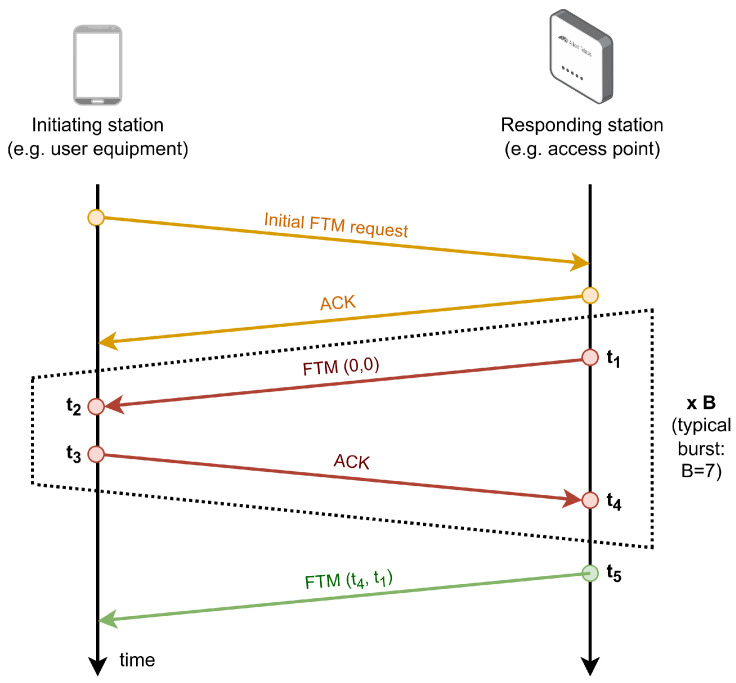
IEEE 802.11mc procedure for the RTT estimation. A typical burst for the Android API comprises seven RTT measurements.

Every fingerprinting solution needs constructing the fingerprint database as a first step. Therefore, a grid of reference points (RPs), where the STA takes measurements, must be defined. Since this study focuses on FTM-based fingerprints in the first place, at each RP, the STA has to run the IEEE 802.11mc procedure with every AP in sight; the estimated distance to each AP is then stored in a database. Therefore, both the STA and the APs need to be IEEE 802.11mc compatible devices. Figure 1 shows the FTM procedure, through which four timestamps are recorded: *t_2_* and *t_3_* for the STA (i.e., the initiating station on the left in Figure 1), and *t_1_* and *t_4_* for the AP (i.e., the responding station on the right in Figure 1). Another frame (e.g., the last FTM frame sent at *t*_5_ in Figure 1) needs to be sent to the STA with the two latter timestamps. Thus, the STA can use the four timestamps to easily compute the RTT, as

(1) $$RTT=[\left(t_{4}-t_{1}\right)-\left(t_{3}-t_{2}\right)].$$

Since RTT estimations generally contain errors (e.g., due to bandwidth limitations, multipath propagation, oscillator drift and jitter [38]), a burst of *B* RTT samples per estimation are usually requested instead of just one. In addition, a calibration function is very often included, which is used to correct the expected measurement errors according to the magnitude of the observation. This is the case for the Android O.S. versions 9 to 11, which returns the average estimated distance over seven RTT samples (i.e., eight FTM messages as shown in Figure 1), which is the typical burst size in the Android API; more recently, the Android 12 release allows the user to set the size of the burst, but the default burst size remains set to 8 FTMs per estimation. In order to compare the RTT-based ranging performance with the RSS-based one of the SoA, the RSS received from every AP at sight and at every RP is also stored in the database.

### 2.2. Experimental Scenarios

**Figure 2:**
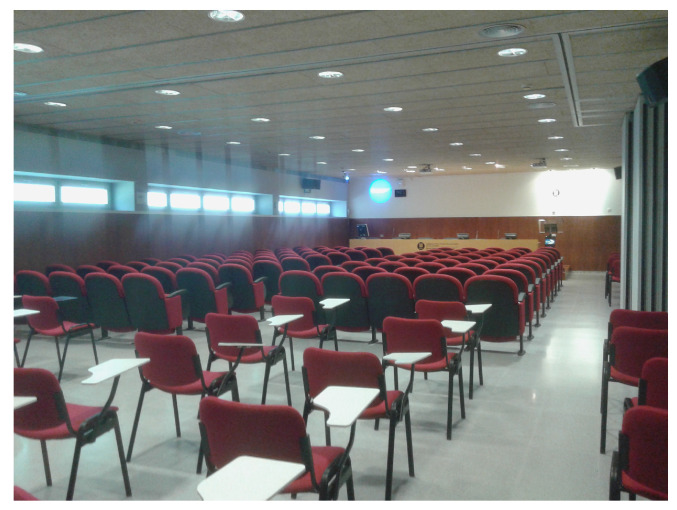
Detailed interior view of the auditorium at EETAC.

The RTT measurements were collected inside the auditorium at the UPC School of Telecommunications and Aerospace Engineering in Castelldefels (EETAC). The scenario is a rectangular area of 19.2 × 8 m, with a few narrow windows and a large number of individual chairs, as shown in Figure 2. A grid of 5 × 5 RPs has been drawn in this area, with a width of 4.8 m on the *x*-axis and 2 m on the *y*-axis. This room is selected to provide a challenging scenario in terms of positioning, as it represents an area with a high density of Wi-Fi networks, where other outdoor solutions (e.g., GNSS) hardly work. In this first analysis, LOS conditions are favored (e.g., no people are allowed in the room during the measurement campaign); in a future analysis, noticeable NLOS conditions will eventually be introduced.

Eight IEEE 802.11mc compatible APs have been set up inside the auditorium. As we could not settle them on the ceiling, they are placed on the available furniture in the room (e.g., on the tables next to the chairs, on the sills, etc., at a height of 1.5 m). The APs were not connected to the internet, just to the power supply. Two AP models are used: the Google AP “Google WiFi” [39], which was one of the few APs that officially supported the technology [26] at the time of the measurement campaign, and the Linksys Velop AC6600 [40], a model that, despite not announcing the FTM capability, is able to run the IEEE 802.11mc procedure. The main configuration parameters for each AP are summarized in Table 2. Both APs work in the ISM bands of 2.4 GHz and 5 GHz (U-NII-1), while the Linksys AP also supports a third band, i.e., U-NII-2C. The U-NII-1 band is utilized for Wi-Fi working indoors (power limited to 200 mW), while the scope of the U-NII-2C band is Wi-Fi networks featuring an augmented transmission power (up to 1000 mW), dynamic frequency selection (DFS) and transmission power control (TPC) features. Nevertheless, the Google APs can only address the FTM frames exchanged in the 5 GHz band, while the Linksys AP is able to handle the FTM frames exchanged in all the frequency bands (see Table 2). A new model of the Google AP has been recently released that is capable of handling FTM frames in the 2.4 GHz band. However, it was not available at the time of the measurement campaign and, moreover, according to Google’s specifications, it does not officially support the FTM procedure in the 2.4 GHz band. A recent work [41] also suggests that the behavior of the RSS and the RTT observables are rather similar at the 2.4 GHz band, which might explain Google’s decision of leaving 2.4 GHz FTMs out of its devices.

**Figure 3:**
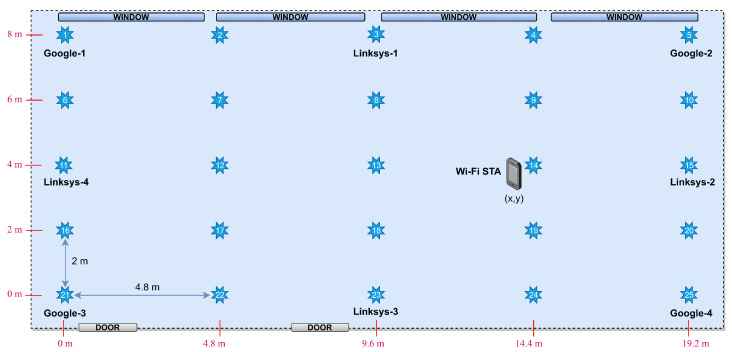
Scenario with four Google and four Linksys APs, and 25 reference points (blue stars).

Four Google APs are placed at the corners of the rectangular area, and the remaining four Linksys APs in a diamond configuration, as shown in Figure 3. The coordinates of every RP are shown in red. Such a symmetric layout is often used as a reference for positioning solutions since it minimizes the geometric dilution of precision (GDOP), alleviating the noise of the positioning model in multilateration solutions, maximizing the entropy of the measured RTT/range fingerprints and aiding in the classification task of fingerprinting solutions.

A Google Pixel 3a phone running Android 10 (Q) is used for data gathering and performance assessment. The application presented in [42] is employed, which uses the reflection capabilities of Java to force collecting data from APs that do not advertise the FTM capability, but support it. Wi-Fi scan throttling is disabled on the device to minimize the time required to gather all the data. The STA is mounted on a tripod with a height of 1 m from the ground, thus emulating the normal placement of a mobile device when held by a user.

**Figure 4:**
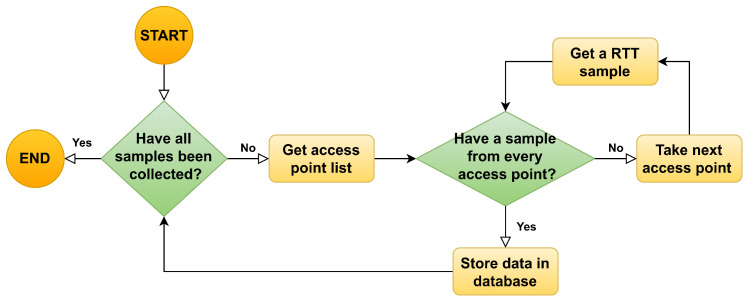
Procedure followed to collect the RTT samples at one reference point in the grid.

The procedure followed by the application in order to collect the data are illustrated in Figure 4. Whenever a data collection campaign is started, the application begins by scanning the radio medium in order to discover the APs at sight. Then, the APs that is not compatible with the IEEE 802.11mc technology are removed from the list, and a sample is collected for every AP that remain in the list. All the collected samples are considered to belong to the same epoch and they are stored in the database accordingly. This procedure is finally repeated until the data for all the requested epochs are collected.

Collecting fingerprinting data are known to be a time demanding task. In this study, approximately 32 h were required to gather all the data (i.e., 100 samples from 8 APs at 25 RPs). There are some approaches that could be taken in order to alleviate the cost of building the fingerprinting database, such as the one proposed in [12], but this is out of the scope of this paper.

## 3. Fingerprint Database Assessment

### 3.1. FTM Observations

At every RP in the grid, 50 RTT samples are gathered with a 5-s delay between samples in order to reduce the time correlation between consecutive fingerprints. This procedure is repeated twice, placing the STA in the two orientations in which it usually works: portrait and landscape. This allows the study of signal reception capabilities under different working conditions and the assessment of their impact on range estimation. Table 3 summarizes the sampling parameters of the measurement stage.

**Figure 5:**
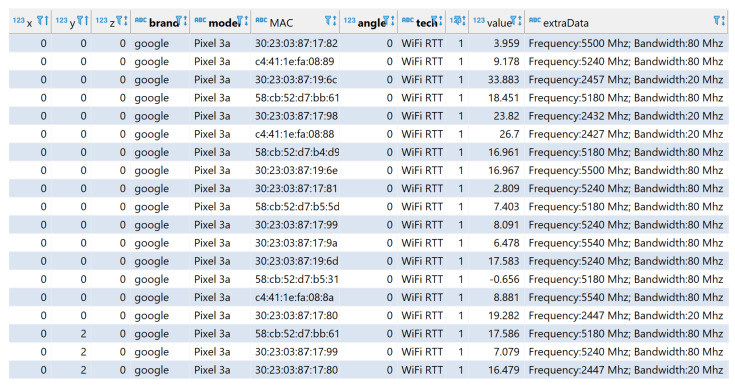
Example of a subset of the features in the dataset made up of “WiFi RTT” measurements. The feature named “value” represents the average distance measured at given positions in the selected scenario.

From this measurement campaign, approximately 20,000 RTT samples are stored in the database. Each sample is the distance reported by the same Android API after averaging the burst of seven RTT samples that the STA has collected from one AP. Although this averaging is expected to reduce the estimation error, still large deviations in the computed distances may be observed if the scenario involves a certain degree of multipath, as can happen here due to the chairs, desks, and ledges. An example of the resulting dataset is depicted in Figure 5, where the selected “technology” is “WiFi RTT” (i.e., RSS observations are filtered out) and the feature named “value” represents the average distance in meters reported by Android. “x”, “y”, and “z” represent the coordinates where the measurement was taken. The brand and model of the STA used for the measurements are given in the feature with the same name, while the APs are identified through the “MAC” parameter (i.e., Google APs are the ones with MAC address starting with 58:cb:52, while the others are Linksys APs). In addition, the orientation (0 or 1) of the STA during the measurement is represented by “angle”. The field “extraData” summarizes the frequency used and other channel characteristics. The whole dataset used in this study is available at [43] for further use.

The ranges at each AP may be refined to remove potential outliers, which generally distort the performance of the fingerprinting position estimation. In this work, a 95% Gaussian window is applied to the data, thus removing 5% of the most unlikely data.

Before exploring how RTT observables can enhance the performance of current positioning systems based on fingerprinting (see Section 4), the quality of the RTT observables such systems would work with is studied. Table 4 shows the mean and standard deviation of the ranging error of all the gathered samples. As shown, observations in the 5 GHz bands underestimated actual distances by 1.9 m in average, which is in line with the results obtained in other studies [6]. Even though the accuracy of the ranging error is quite low, which means biased samples, the standard deviation is fairly good. From the point of view of an observable, this is essential, since bias can be removed introducing calibration stages, while facing standard deviation is much challenging. The best performing AP has a standard deviation of approximately 85 cm, and the worst AP has a standard deviation of approximately 134 cm. Although the average ranging error of the measurements taken in the 5 GHz band is similar (i.e., Google-Indoor, Linksys-Indoor, Linksys-DFS), indoor channels proved to provide more stable RTT measurements than those collected in the DFS band. Furthermore, the Linksys APs seems to provide more refined ranges if compared with those coming from Google APs. This is interesting, since Google APs officially supports the IEEE 802.11mc technology, whereas Linksys APs do not.

As already observed in other studies [30,42], the distances observed in the 2.4 GHz band are always far less accurate than those observed at 5 GHz, regardless of the AP vendor and channel. In addition, the author in [44] suggests that the accuracy of the observations depends roughly inversely on the bandwidth of the signal. These inaccurate estimations at 2.4 GHz discourage their use in multilateration positioning algorithms [41,42], at least when working with a 20 MHz bandwidth as in this study. Moreover, a multilateration approach would require removing the bias observed in the measurements, which depends on both the equipment used (i.e., both the user terminal and the AP) and the frequency channel [44]. In this paper, a different approach is explored (i.e., fingerprinting) for which the uniqueness of the measurements at each RP is more important than their accuracy. Therefore, fingerprinting can cope with biased estimations without requiring a preliminary calibration, whereas this latter is mandatory when multilateration algorithms are run. Accordingly, the 2.4 GHz band is further considered in this study.

**Figure 6:**
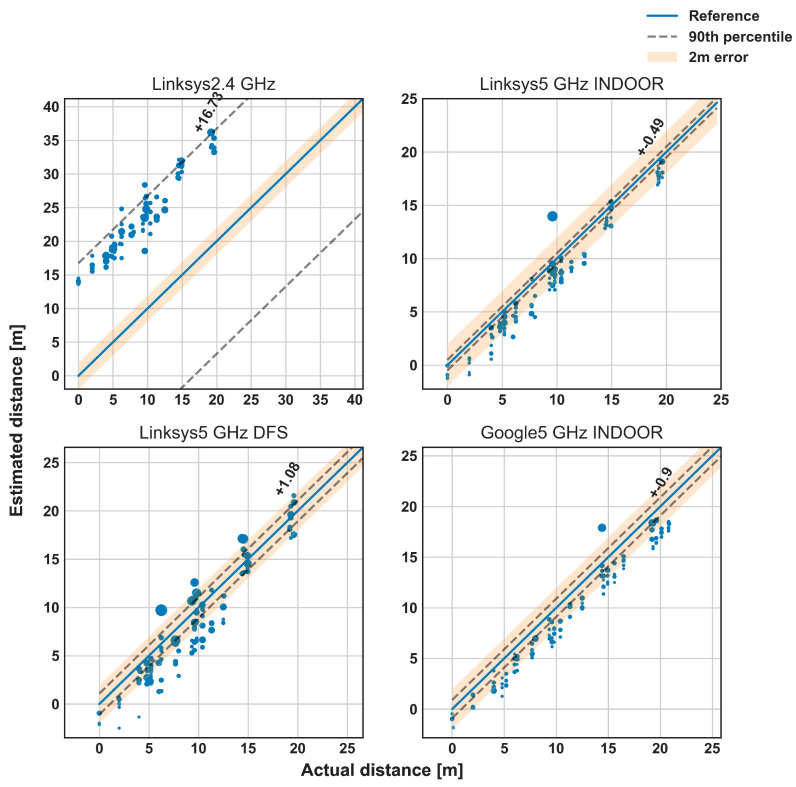
Estimated distance versus actual distance (in meters). The bigger the marker, the higher the standard deviation of the error. The 90th percentile is displayed with the dash-gray line, while the 2-m error in yellow.

Figure 6 provides more details on the RTT observations by depicting the estimated distance as a function of the real distance, for each AP brand and frequency channel. The bigger the marker, the higher the standard deviation of the error between the estimated and the real distance. The blue line represents the ideal situation where the estimated and real distances are the same. The 2-m error is also highlighted in yellow for reference. In most of the cases at 5 GHz, the estimated distance is no more than 2 m away from the real distance. The 90th percentile of the overall absolute error is also displayed in Figure 6 as a discontinuous gray line, and it is smaller in the U-NII-1 channel (5 GHz indoor) compared to U-NII-2C (5 GHz DFS), showing higher variability in the latter. In addition, there is no lineal dependency of the error with the distance: the magnitude of the error is similar whatever the distance. At some distances, the standard deviation is higher since there may be hard obstacles in the LOS between the RP and some APs (e.g., at almost 15 m from the Google); in addition, the effects of such obstacles may depend on the frequency channel (e.g., higher standard deviation (STD) at almost 10 m from the Linksys in the U-NII-1, and at 6 and 15 m for the Linksys in the U-NII-2).

**Figure 7:**
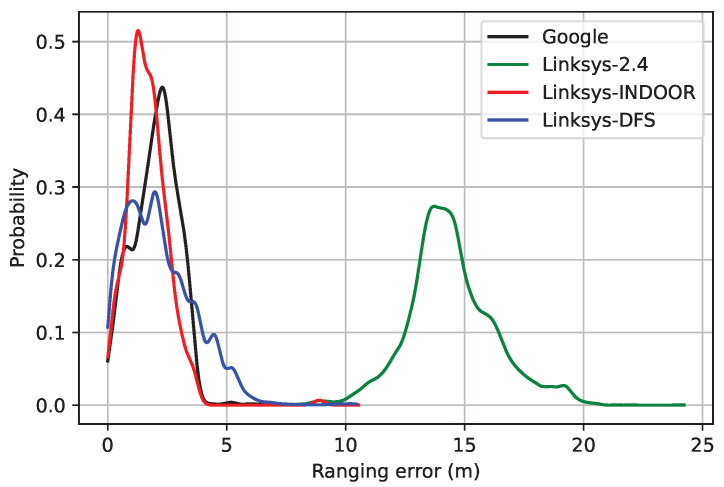
PDF of the ranging error.

The probability density function (PDF) of the absolute value of the ranging error is depicted in Figure 7. The ranging error follows a right-skewed multimodal distribution made of several Gaussian-like components, as already found in previous studies, both in LOS [42] and NLOS [30] conditions. Moreover, if the main component of the error is wider, the modes are higher; conversely, the lower the standard deviation, the more likely the error looks like a unimodal distribution, as it is for the ranging error in the U-NII-1 band (i.e., Google and Linksys-Indoor).

### 3.2. Measurements

**Figure 8:**
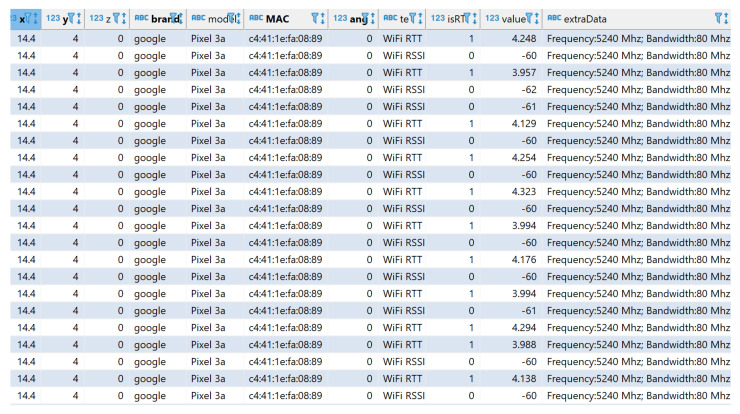
Variability of the observations at one single RP to a single AP, both in the case of “WiFi RTT” and “WiFi RSS” measurements. The feature named “value” represents, in the former, the average distance in meters and, in the latter case, the RSS in dBm.

In order to compare the behavior of the RTT-based fingerprint with the SoA RSS-based one, the RSS at each RP and from every AP was also collected and stored in the database. A portion of the whole dataset, with both FTM-based and RSS-based measurements, is depicted in Figure 8, where the feature named “value” represents the average distance in meters reported by Android in the case of “WiFi RTT”, or the RSS in dBm in the case of “WiFi RSSI”. One single RP (“x”, “y” and “z”), one single AP (“MAC”) and one single orientation (“angle”) are selected in order to show the variability of the reported measurements due to e.g., multipath. In order to better characterize the behavior of the estimations even in a static environment (i.e., nobody was admitted in the room while taking measurements), several samples (e.g., 50 per orientation in this study) were gathered. The statistics for the RTT-based estimations are reported in Section 3.1.

The stability of the RSS observations is provided in Table 5, where the average, minimum, and maximum STD of the RSS observations are displayed. The Linksys-DFS APs register the highest average STD, while the Google APs the lowest one. The smallest minimum STD is observed in the 2.4 GHz band with a decrease of more than 76%, while the highest maximum STD is reported by the Google APs with an increase of more than 135%. The Linksys-DFS is the one with the smallest deviations from the average STD, despite the latter being the highest among all the APs. Even though we cannot directly compare the RTT and RSS observations, since the former provides a raw estimation of the ranging performance while a ranging model must be applied to the latter in order to infer the resulting distance estimations, yet, as depicted in Table 6, the minimum and maximum deviations over the average STD in the case of the RTT observations are smaller if compared to the case of the RSS ones. The Linksys-Indoor APs have the lowest average STD and the smallest deviations over the average (−29% and +40%), while the Google APs report the highest deviations over the average.

## 4. Performance of the FTM-Based Positioning Model

**Figure 9:**
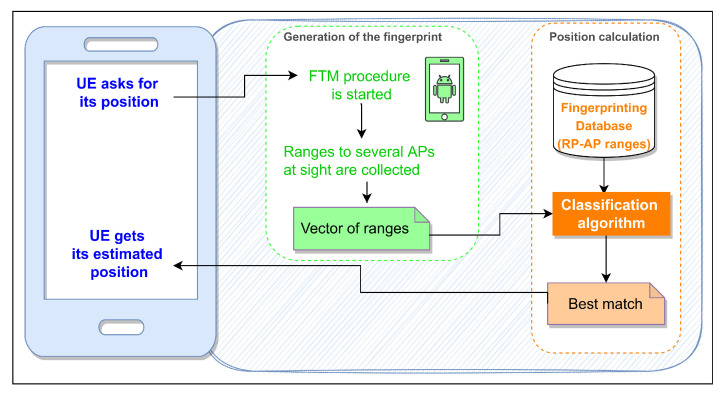
Flowchart of the FTM-based positioning model.

The aim of the positioning model is to convert fingerprints reported by STAs into positions. When a STA asks for its position, the FTM procedure is started, and the Android API returns the average distances calculated with each AP in sight, yielding a fingerprint vector of ranges. Then, a ML classification algorithm is run, so the fingerprint (or combination of fingerprints) in the database that best matches the input fingerprint vector is reported. Finally, a position is computed from the locations associated with the set of reported fingerprints. A distance model, which works as a loss function, is used to quantify the quality of the match: the larger the value is, the worse the position estimation. This procedure is summarized in Figure 9.

The core of the positioning model is the classification algorithm that is used to compute the position. Fixing the user’s position in fingerprinting systems can be understood as a classification problem, where RPs are the classes, and the ranges or RSS to each AP are the features. Several classification algorithms can be used for positioning. Although the NN is considered as one of the best solutions for RSS-based fingerprinting [32], it is not clear whether this statement also applies to fingerprinting solutions that use the RTT. Hence, a wide set of classifiers that are typically used in other fingerprint-based positioning works [34] have been considered in this study, including Weighted K-nearest neighbor (K = 3) (WKNN-3), NN, random forest (RF), extra tree (ET), AdaBoost (AB), and support-vector machine (SVM).

In the following sections, the performance of the FTM fingerprint-based positioning is compared with SoA RSS-based one; the latter is always depicted with dashed lines in the following figures. We start with a scenario with optimal coverage, i.e., with four APs of the same vendor and where the best performance is expected (Section 4.1). Moreover, since FTM estimations consume bandwidth to regular data transfer in the shared WiFi spectrum, we also aim at assessing the impact of using less than 4 APs on the performance of the positioning model (Section 4.2). Furthermore, the scarce amount of devices currently supporting the IEEE 802.11mc technology makes the study of the RTT contribution to fingerprinting solutions especially interesting in the case of scenarios with a reduced amount of available APs.

It should be noted that the results shown in the following sections are tested on the same RPs where the RTT/RSS measurements are taken and where the dataset is constructed; 20% of the whole dataset has been reserved for testing purposes. The point of making the test space discrete is to assess the performance of classification algorithms under well known conditions. Further filtering on resulting positions (e.g., basic centroid [35] and particle filter [36], etc.) can improve the figures presented in this work when measurements are taken anywhere in the location area. However, the goal of this work is not providing a complete location system definition, but focusing on assessing the contribution of FTM observables to fingerprinting solutions. Thus, the raw performance of the classification when applied to FTM observables is assessed and compared to what RSS-based fingerprinting solutions would achieve under the same conditions. The accuracy of the classifiers and how much the classification mismatch impacts the positioning error is then evaluated under both approaches, i.e., RTT and RSS-based fingerprinting solutions, so that the use of the RTT as an observable can be validated, either coupling to already proposed RSS-based systems [9,45] or being used as the main observable for further fingerprint solutions.

Therefore, the application and further assessment of these upper-layer algorithms has been postponed for future work.

### 4.1. Optimum Coverage

**Figure 10:**
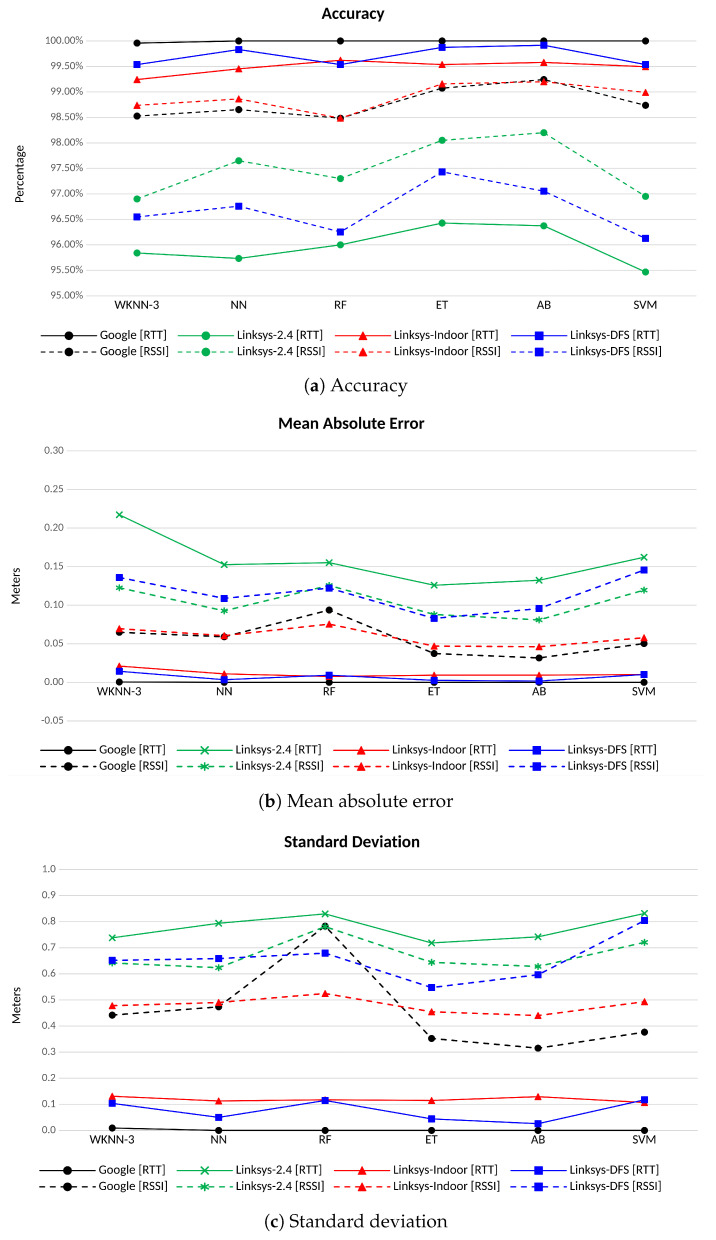
Accuracy, mean absolute error and standard deviation for different ML algorithms, APs vendor and channel. FTM-based in solid lines, RSS-based in dashed lines.

Figure 10a shows the accuracy achieved by each classifier when estimating the position for each AP vendor and frequency channel. The accuracy measures the goodness of the match, that is, how probable it is that the estimated position (most probable RP) matches the real position. All the algorithms achieve very good performance, with an accuracy that is higher than 95% for all the APs and channels. However, except for the Linksys in the 2.4 GHz band (in green in the picture), the FTM-based positioning (in solid lines) always outperforms the RSS-based, achieving an accuracy that is always higher than 99%. As shown in Figure 7, the range errors of the estimations taken at 2.4 GHz are noticeably noisier than those at 5 GHz, since the 2.4 GHz band allocates much more transmissions from multiple technologies that compete for narrow band channels. Therefore, the advantages of precise time measurements for fingerprinting over observations of the RSS taken in this band disappear. Indeed, several user devices and APs often restrict the FTM procedure to the 5 GHz band only (e.g., Google APs). The Google APs (in black in Figure 10a) always achieve the highest accuracy (i.e., 100%) regardless of the ML algorithm. Considering the Linksys APs, better accuracy is always achieved when using the Linksys-DFS AP (in blue) compared with the Linksys-Indoor AP (in red), even though differences between them can be considered negligible. Despite the poor performance in terms of ranging accuracy (see Table 4), the classification accuracy, when the Linksys APs in the 2.4 GHz band (in green) is used, is still higher than 95%.

Accuracy by itself does not provide enough information to guess how wrong the misclassification is. Therefore, the mean average error (MAE) and the STD of the positioning error have been used in this study to illustrate the impact of misclassifications on the positioning error. The MAE provides a mean value that helps to determine how well the classification is performing on average and allows an easy comparison among the performance of different classifiers. On the other hand, the STD provides valuable information on the variability of the positioning error depending on the RP.

Focusing on the MAE, a similar behavior as the one shown in terms of accuracy is observed. The error in the positioning is evaluated as the Euclidean distance between the RP estimated by the classification algorithm and the RP corresponding to the validation fingerprint. Thus, when the classification algorithm provides the correct match (i.e., the two RPs are the same), the error is 0. When the match is not correct, the minimum error that is observed will be 2 m, which is the lowest distance between RPs in the grid according to our scenario in Figure 3. Figure 10b shows the MAE between the estimated and the real distances of the FTM-based positioning (solid lines) and of the RSS-based positioning (dashed line). Again, all the algorithms always achieve a MAE of less than 25 cm, and even less than 15 cm if the measurements taken at 2.4 GHz are excluded. Except for the latter, the MAE of the FTM-based positioning always outperforms the one obtained with RSS measurements. Similarly, the STD of the positioning error (Figure 10c) is always lower than 0.25 m for all the FTM-based positioning in the 5 GHz bands. Conversely, when the RSS observables are used, the STD of the positioning error raises up to 0.83 m, which means that the RSS is prone to higher positioning errors when mismatching, if compared to FTM-based solutions. On the overall, ET seems to provide the best results in terms of precision for all the APs and frequency bands.

This work presents the results obtained using fingerprints from two orientations. It has been observed that the use of fingerprints from different orientations impacts the performance of the classifiers, since the classification space grows and so does the chance for a misclassification. When fingerprints in only one orientation are used, the performance observed is maintained for different frequency bands and different devices, no matter which orientation is considered. As an example, more than 99.9% of accuracy is achieved in the 5 GHz Indoor channels when only one orientation is used, independently of the observable used (i.e., RTT or RSS) and the applied classifier; this result is slightly better than the one shown in Figure 10a when two orientations are considered. However, it is important to take into account that, in real deployments, the user may keep the device in any orientation, thus making important to observe how the proposed solution copes with this situation.

### 4.2. Limited Coverage

In the previous section, the performance of the different ML algorithms fed with RSS or RTT observables has been shown when all the APs of a given vendor and frequency channel (i.e., four Google APs, four Linksys APs at 5 GHz Indoor, etc.) are considered. In the following, we aim at assessing the performance when considering a lower number of APs of a given vendor and frequency band. The number of measurements that are needed in order to gather a pretty accurate estimation of the position is fundamental for two main reasons: firstly, in the case of the FTM-based positioning, the FTM-procedure needs the STA and the AP to explicitly exchange special messages in order to obtain the timestamps and, then, the distance estimation. The more the APs to be pinged, the higher the overhead and, thus, the lower the available bandwidth for other users’ data exchange. This scalability issue is one of the main drawbacks of the FTM-based solution proposed in the IEEE 802.11mc and the reason behind our proposal of the FTM- fingerprint-based approach in this paper. Secondly, the availability of APs that support the IEEE 802.11mc procedure is relatively scarce. Therefore, it is not likely to find scenarios with a large number of RTT-able APs in sight, as shown by a recent wardriving campaign conducted in the city of Barcelona in the framework of the BANSHEE project [46] in search for FTM-able devices [47].

Thus, several scenarios have been generated by disabling some of the APs initially deployed. The switching off sequence followed in this study to set up those scenarios is reported in Table 7. Scenario “3 APs”, when the Google APs are considered, refers to the RTT (RTT-3 APs) or the RSS (RSS-3 APs) measurements gathered from Google 2, Google 3, and Google 4; similarly, scenario “2 APs” refers to the measurements from Google 2 and Google 3, and scenario “1 AP” uses the measurements from Google 3 only. In the case of the Linksys APs, scenario “3 APs” refers to Linksys 1, Linksys 2 and Linksys 3, scenario “2 APs” to Linksys 1 and Linksys 2, and scenario “1 AP” to Linksys 2.

**Figure 11:**
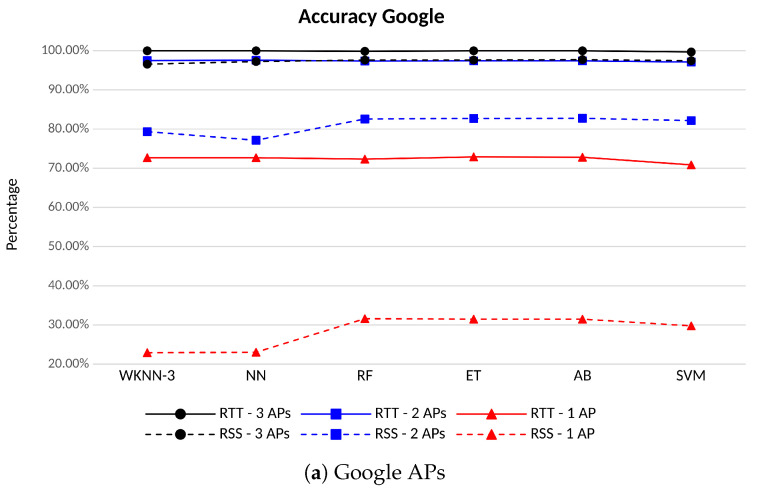

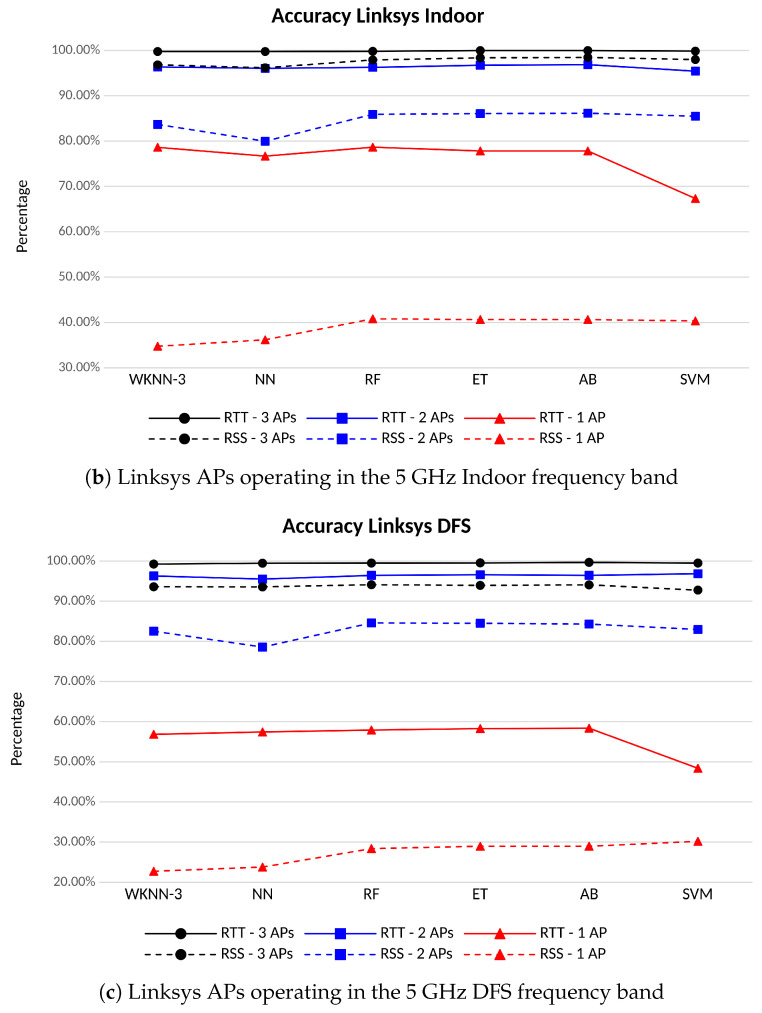
Accuracy for different ML algorithms and number of APs of the same vendor and channel. FTM-based in solid lines, RSS-based in dashed lines.

Figure 11 shows the accuracy for the different scenarios (e.g., 3 APs in black, 2 APs in blue, 1 AP in red) and when different ML algorithms are run. The results for the Google APs are displayed in Figure 11a, in Figure 11b for the Linksys at 5 GHz Indoor, and in Figure 11c for the Linksys at 5 GHz DFS. The behavior is very similar for the three cases: the best performance is always achieved when employing the RTT measurements (continuous lines), with an accuracy that is always more than 99.2% with 3 APs (in black), and it stays between 95.4% and 97.6% with 2 APs (in blue), for all the ML algorithms considered in this study. This latter result is particularly important, as any multilateration approach would need at least the measurements from three APs to obtain the position, while we are proving here that, with a fingerprinting approach, more than 95% of accuracy is still obtained even with 2 APs. When considering 1 AP only (in red), the accuracy drops below 79% and shows more variability depending on the ML algorithm and the channel: the SVM achieves the lowest accuracy for all the APs (70.8% for the Google, 67.3% for the Linksys Indoor, and 48.4% for the Linksys DFS). In addition, in general, the Linksys DFS always shows the lowest accuracy, whatever the classifier, compared to the other channels, thus the impact of considering scenarios with less APs is higher in this channel.

On the other hand, the accuracy obtained with the RSS-based measurements is always smaller than the one achieved with the RTT. The best performance is obtained with the Linksys Indoor for all the scenarios, with values between 96.2% and 98.4% with 3 APs (in black and dashed line), between 80.0% and 86.1% with 2 APs (in blue and dashed line), and between 34.7% and 40.8% with 1 AP (in red and dashed line). The results show that the difference in the accuracy between RTT-based and RSS-based solutions grows as the amount of APs drops. Thus, RTT observables may effectively assist classical RSS fingerprinting solutions in scenarios where just few APs are in sight.

**Figure 12:**
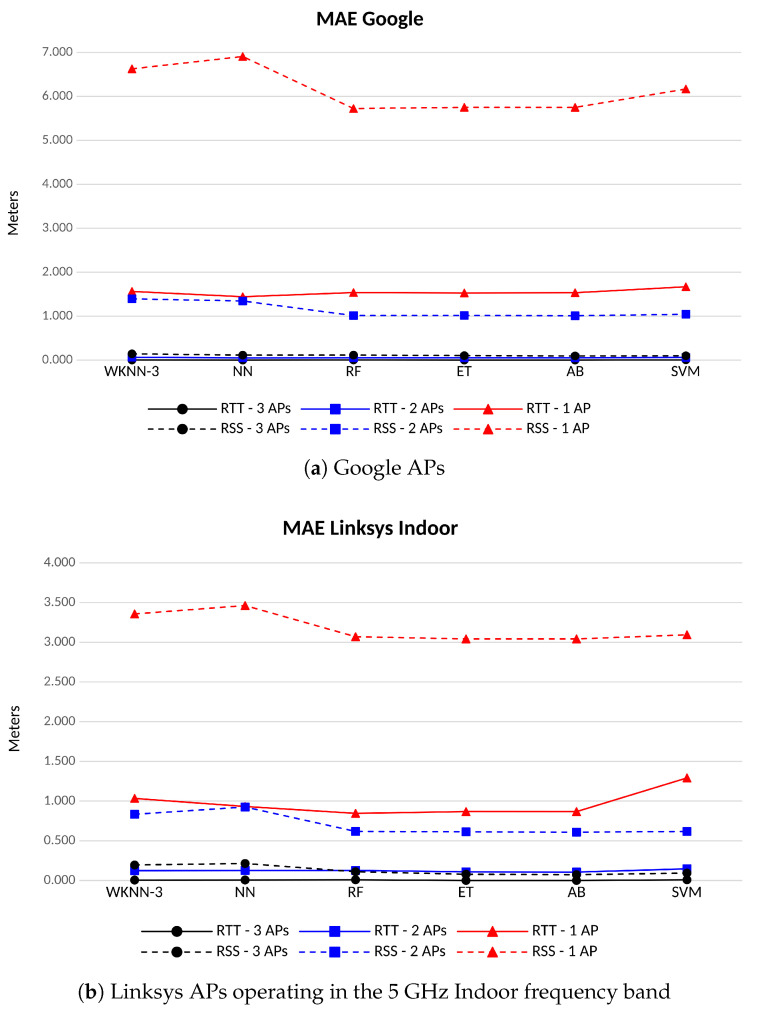

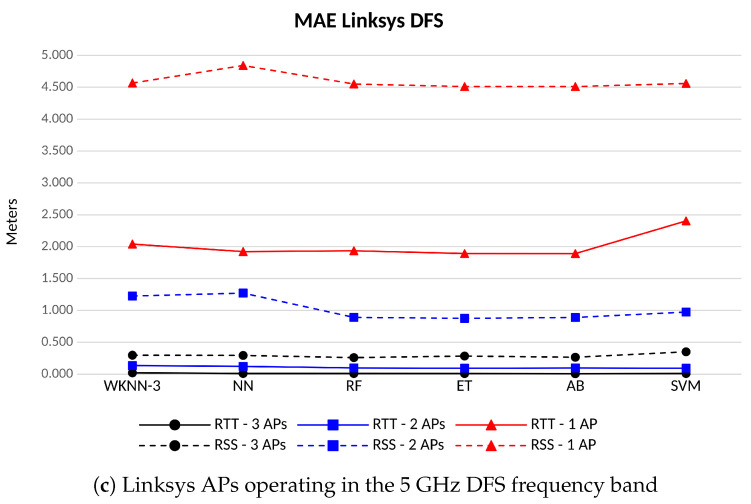
MAE for different ML algorithms and number of APs of the same vendor and channel. FTM-based in solid lines, RSS-based in dashed lines.

Figure 12 shows the MAE of the position for the different scenarios and when different ML algorithms are run. In the scenarios with more than 1 AP, using the RTT as an observable yield to positioning errors of a few centimeters (between 2 and 6), whatever the AP brand and frequency band. In the case of a single AP in sight, Linksys APs working in the indoor 5 GHz band are the best performing ones, with an MAE of less than 87 cm. Google APs provide an average positioning error close to 1.5 m, which almost doubles what is achieved by Linksys in the indoor channel. However, Linksys APs using the DFS band involve average positioning errors noticeably larger than the others, reaching figures very close to the 2 m. Therefore, mismatching in this scenario with only one Linksys AP in the DFS channel involves larger errors than in those using indoor channels. Linksys APs provide better results in RSS classification than Google’s. In the case of the RSS, the DFS band eases the classification and yield to better performance in terms of positioning error. Anyway, the RSS reveals itself as a poorer observable in terms of classification, leading to larger MAE figures than those achieved with the RTT. As in the case of the accuracy, the lower the number the APs in sight, the higher the positioning error and the larger the difference between the performance achieved by RTT and RSS solutions.

Globally, the best performing classifiers are the RF, ET, and AB. NN-based algorithms seem to work fine when the amount of observables is high, but they faint when there are only one (or two in the case of RSS) APs in coverage.

**Figure 13:**
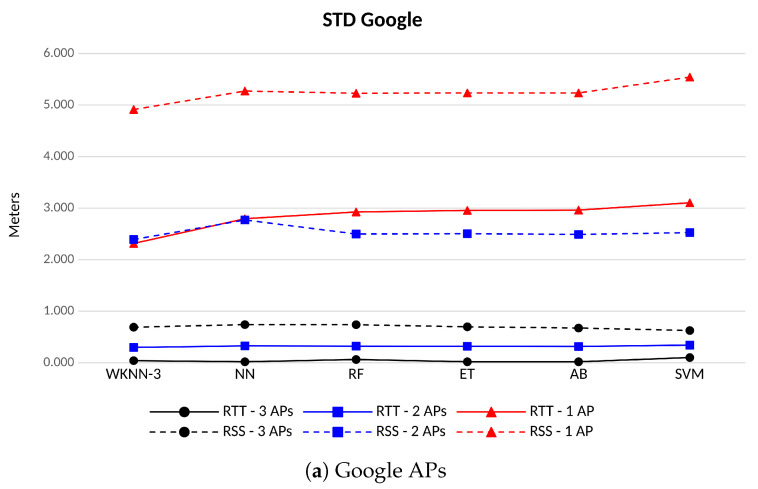

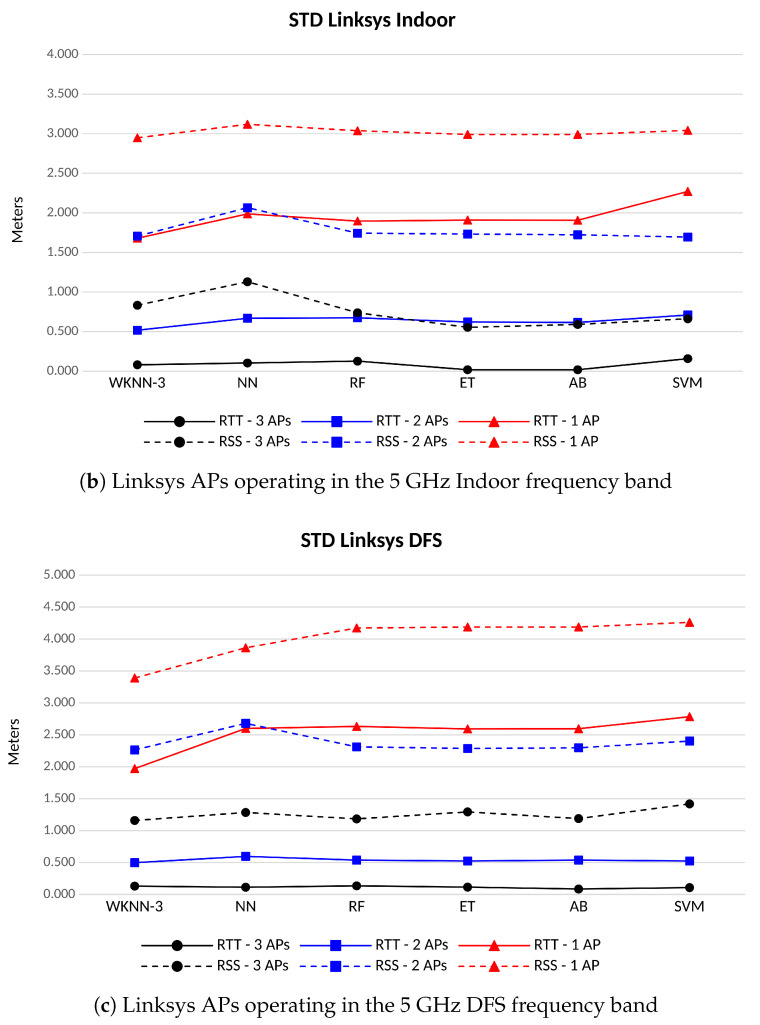
Standard deviation of the positioning error for different ML algorithms and number of APs of the same vendor and channel. FTM-based in solid lines, RSS-based in dashed lines.

The standard deviation of the position error is displayed in Figure 13. When RTT observables are used, Google APs provide the lowest STD of the position error in scenarios with at least two APs in sight (in blue), with values below 33 cm; on the other hand, the positions computed using Linksys APs tend to be noisier. In this latter case, Linksys APs perform similarly whatever the band used. In the scenario with only one AP in coverage (in red), results are inverted and Linksys APs perform better than Google’s, especially when indoor channels are used: STD of 1.7 m (Linksys indoor) and 2.3 m (Google). Positions coming from RSS observables are clearly noisier than those computed from RTT/range estimations, whatever the number of APs in sight. Indeed, most of the time the STD of positions computed fromthe RSS doubles the values achieved with the FTM estimations. In terms of classification algorithms, WKNN-3 reveals itself as the most performing one in scenarios with low coverage (i.e., 2 or less APs in sight), while in scenarios with better coverage, ET and AB provide more stable positioning errors if compared with the remaining algorithms.

## 5. Conclusions

Fingerprint-based positioning in Wi-Fi has been historically relying on RSS observations. However, since FTM frames were introduced by IEEE 802.11mc, precise timestamps are also a potential source of information for fingerprinting solutions. This work is one of the firsts in the literature that studies the benefits of using RTT/range observations instead of the classical RSS approach in fingerprint-based positioning systems. Several ML classification algorithms, typically used for RSS- fingerprint-based positioning, are assessed in this paper with observations based on IEEE 802.11mc FTM. The classification space has been reduced to a discrete amount of RPs, as fingerprinting solutions tend to follow this approach for computational sake.

Results show that in over-determined scenarios (i.e., with a large amount of APs), the performance of fingerprinting is similar whatever the source of information (i.e., RTT or RSS), although the former performs slightly better. Nevertheless, in scenarios with a reduced amount of APs, the use of the RTT noticeably improves both the accuracy of the ML algorithms used for classification and the positioning error of the returned locations, if compared with the performance of regular RSS-based fingerprinting solutions. This finding is very important also in light of providing a solution to the well-known scalability problem faced by the FTM procedure: while the multilateration approach cannot work with measurements from less than three APs, the FTM-fingerprint-based approach proposed here can cope with these under-coverage scenarios, achieving more than 95% of accuracy and a precision of less than 6 cm even with only two APs in the assessed scenarios.

The best performing algorithms, both in terms of accuracy and positioning errors, are the ET and AB and, to a lesser extent, the NN-based algorithms, especially the WKNN-3. These two latter algorithms provide acceptable estimations for the Google APs, while with the Linksys APs the performance degrades, especially in terms of the MAE. However, their simplicity and lower computational requirements, compared with ET or AB, encourage their use in well-covered scenarios.

The ET and AB prove to be the best choice in under-covered scenarios, with an accuracy of at least 73% and 77% when Google and Linksys APs are used, respectively. The maximum absolute mean error is about 90 cm when RTT observables are used with a single Linksys AP working in the 5 GHz indoor band. Results in the U-NII-2C band show higher positioning errors on average, which often doubles that reported in the indoor band. Hence, the use of measurements taken in the DFS channel is discouraged in pure RTT fingerprinting systems; instead, they can be used to complement the already deployed RSS-based ones.

Considering the coarse-grained grid used in this work, for which an error in the classification represents a minimum error of 2 m, the observed average errors provided by the ET, AB, and NN-based algorithms, when using less than four APs, are still small. Furthermore, the results demonstrate that a classification mismatch involves estimated positions closer to the actual ones, which means smaller than 1-σ errors, and hence more accurate positioning solutions once calibrated.

In addition, considering that the higher the number of APs needed for accurate positioning the lower the available throughput for normal IEEE 802.11 operation, we can conclude that the use of ET and AB with a subset of the APs in sight paves the way for scalable fingerprinting solutions when the RTTs are used as an observable.

Accordingly, RTT-assisted fingerprinting solutions raise a valuable solution for achieving a sub-meter accuracy level, even in harsh scenarios with only two APs in sight, which fits the requirements of most of the current indoor location based services (e.g., navigation, tracking, intelligent storage systems, etc.) and opens a promising research path in the near future.

## 6. Future Work

Results have demonstrated that the RTT is a valuable observable and that it can be used in fingerprinting systems to improve the performance of traditional RSS-based ones. However, the RTT is not clearly better than the RSS in all scenarios, as both come with some advantages and drawbacks (see Table 1).

In addition to the accuracy and positioning errors, for which the choice of the observable can be of key importance, fingerprinting systems still have several open issues that the research community is currently addressing. One of them is the cost of calibration, i.e., the offline stage during which the fingerprinting database is built. Some proposals have been presented to alleviate this time-demanding task, most of them consisting of reducing the fingerprinting survey to just few RPs and then interpolating the remaining data [12]. This interpolation is quite hard to achieve when the RSS is used, since radio models are quite complex and deeply bound with the environment where the fingerprinting is going to be deployed. However, the ideally linear dependence of the RTT with the distance is expected to simplify interpolation and represents a great opportunity for a quick and easily maintainable fingerprinting database generation.

Accordingly, there is not a unique observable to be used for fingerprinting systems whatever the conditions. Instead, coupling both observables and trying to obtain the best of each, as proposed in [48], opens a new research path to be explored in the near future.

The impact of the NLOS and of the environment changes in the fingerprinting database are open issues that require further research. Although RTT fingerprinting systems are expected to be less sensitive to NLOS, assessing the capabilities of simple algorithms to properly classify fingerprints under complex reception conditions is mandatory for a proper evaluation of the location solution. The same applies to environment changes, which have been considered a great challenge so far for traditional RSS fingerprinting systems; their impact requires further study also for RTT-based solutions. We aim at studying the impact of both issues in the near future in order to demonstrate the capabilities of the RTT to complement or replace the RSS in the fingerprint-based positioning solutions of the future.

## Figures and Tables

**Table 1:** Properties of RTT and RSS as observables for fingerprint-based positioning.

Parameter	RTT	RSS
System scalability	Medium	High
Observable availability	Low	High
Observable stability	High	Low
Environment sensitiveness	Medium	High
Database estimation complexity	Medium	High
Device calibration	Medium	High

**Table 2:** AP configuration parameters.

Parameter	Google AP	Linksys Velop
Bands supporting IEEE 802.11mc	5 GHz	2.4 and 5 GHz
2.4 GHz channel bandwidth	20 MHz	20 MHz
2.4 GHz channel number	11	11
5 GHz channel bandwidth	80 MHz	80 MHz
5 GHz channel number	42 (U-NII-1)	42 (U-NII-1)
		106 (U-NII-2C)

**Table 3:** Sampling parameters.

Parameter	Value
Device	Google Pixel 3a
O.S. version	Android 10.0 (Q)
Orientation	Portrait and landscape
Samples per orientation	50
Time between consecutive samples	5 s
STA height from the floor	1 m
Wi-Fi AP height from the floor	1.5 m

**Table 4:** Mean and standard deviation of the ranging error in the assessed scenarios.

AP	Band	Mean	STD
Google-Indoor	5 GHz (U-NII-1)	−2.004 m	0.881 m
Linksys-2.4	2.4 GHz	14.332 m	1.149 m
Linksys-Indoor	5 GHz (U-NII-1)	−1.690 m	0.848 m
Linksys-DFS	5 GHz (U-NII-2C)	−1.799 m	1.335 m

**Table 5:** Stability of the RSS observations (minimum, maximum, and average standard deviation) for each AP vendor and frequency channel.

AP	Band	Avg. STD	Min. STD	Max. STD
Google-Indoor	5 GHz (U-NII-1)	5.889 dB	1.662 dB (−71.77%)	13.864 dB (+135.42%)
Linksys-2.4	2.4 GHz	6.023 dB	1.421 dB (−76.40%)	11.804 dB (+95.98%)
Linksys-Indoor	5 GHz (U-NII-1)	6.352 dB	2.826 dB (−55.51%)	12.273 dB (+93.21%)
Linksys-DFS	5 GHz (U-NII-2C)	6.535 dB	3.190 dB (−51.18%)	11.822 dB (+80.90%)

**Table 6:** Stability of the RTT observations (minimum, maximum, and average standard deviation) for each AP vendor and frequency channel.

AP	Band	Avg. STD	Min. STD	Max. STD
Google-Indoor	5 GHz (U-NII-1)	5.216 m	0.745 m (−85.71%)	8.249 m (+58.14%)
Linksys-2.4	2.4 GHz	5.375 m	3.713 m (−30.92%)	7.751 m (+40.22%)
Linksys-Indoor	5 GHz (U-NII-1)	4.849 m	3.426 m (−29.34%)	6.804 m (+40.31%)
Linksys-DFS	5 GHz (U-NII-2C)	5.355 m	2.908 m (−45.69%)	7.395 m (+38.09%)

**Table 7:** Sequence followed for switching-off the APs.

Number of APs	Disabled APs
3	Google 1, Linksys 4
2	Google 1, Google 4, Linksys 3, Linksys 4
1	Google 1, Google 2, Google 4, Linksys 1, Linksys 3, Linksys 4

## Abbreviations

The following abbreviations are used in this manuscript:

2d	2D	Two dimension
3d	3D	Three dimension
ab	AB	AdaBoost
ack	ACK	Acknowledgement
aoa	AOA	Angle of arrival
ap	AP	Access point
ble	BLE	Bluetooth low energy
csi	CSI	Channel state information
cots	COTS	Commercial/consumer off-the-shelf
dfs	DFS	Dynamic frequency selection
dnn	DNN	Deep neural network
et	ET	Extra tree
ftm	FTM	Fine time measurement
gdop	GDOP	Geometric dilution of precision
gnss	GNSS	Global navigation satellite system
hgb	HGB	Histogram-based gradient boosting classification tree
ism	ISM	Industrial, scientific and medical
los	LOS	Line of sight
mae	MAE	Mean average error
ml	ML	Machine learning
nn	NN	Nearest neighbor
nlos	NLOS	Non-line of sight
pdf	PDF	Probability density function
rf	RF	Random forest
rfid	RFID	Radio frequency identification
rmse	RMSE	Root mean square error
rp	RP	Reference point
rss	RSS	Received signal strength
rtt	RTT	Round trip time
soa	SoA	State of the art
sgd	SGD	Stochastic gradient descent
sta	STA	Station
std	STD	Standard deviation
ss	SS	Service set
svm	SVM	Support-vector machine
tof	TOF	Time-of-flight
tpc	TPC	Transmission power control
uwb	UWB	Ultrawide band
vrp	VRP	Virtual reference point
wifi	Wi-Fi	Wireless Fidelity
wknn	WKNN-3	Weighted K-nearest neighbor (K = 3)
